# Supplementary material for: Location-dependent threat and associated neural abnormalities in clinical anxiety
Source: Commun Biol. 2021 Nov 4;4:1263. doi: 10.1038/s42003-021-02775-x (PMC8568971; doi:10.1038/s42003-021-02775-x)
Supplement: Supplementary file 2 — Supplementary Information [file 42003_2021_2775_MOESM2_ESM.pdf]

Supplementary Information

Environmental navigation. *Overall approach navigation:* Overall approach navigation was measured as participants navigated the environment before collecting a flower. A 2x2x4 ANOVA (zone by group by block) showed a main effect of time with navigation time decreasing from block 1 to block 4 ($F(3,47)=23.06$, $p=2e-12$). No other significant main effects or interactions were observed (F's<2, p's>0.05).

Active approach navigation: Active approach navigation was measured as participants actively navigated towards a flower. A 2x2x4 ANOVA (zone by group by block) showed a main effect of time with navigation time decreasing from block 1 to block 4 ($F(3,47)=13.87$, $p=1e-6$). No other significant main effects or interactions were observed (F's<2, p's>0.05).

Overall task duration: Overall task duration was measured as the total time participants took to complete the task. An independent samples t-test showed no group differences on completing the task ($t(49)=0.42$, $p=0.51$).

Supplementary figures

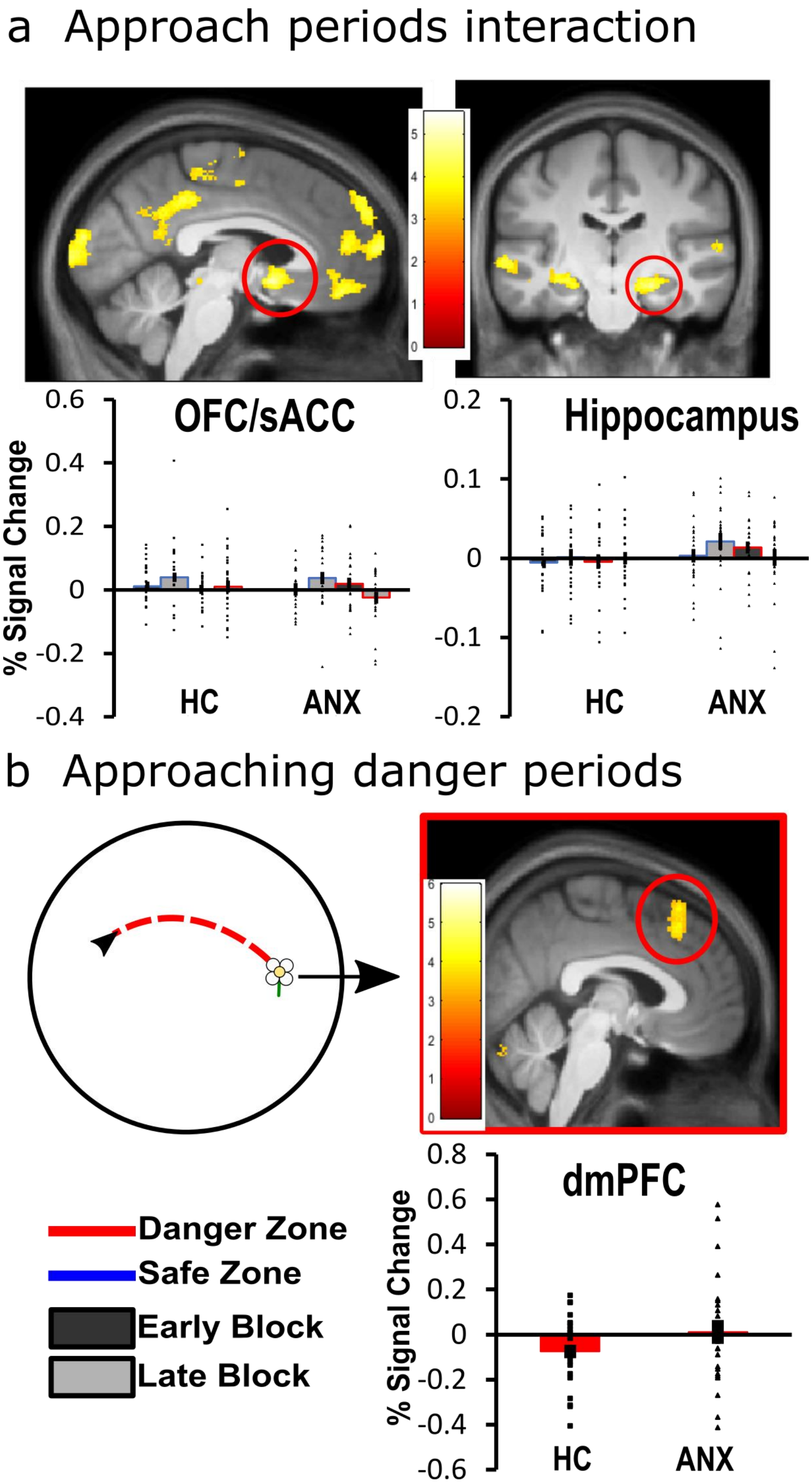

Supplementary Figure 1. fMRI results of approaching flowers. *(A) Zone by block by group interaction shows two opposing patterns of activation between groups in the posterior cingulate cortex (PCC; p<0.05 FWE), ventromedial prefrontal cortex (vmPFC), orbitofrontal cortex/subgenual anterior cingulate cortex (OFC/sACC; top left panel), and anterior hippocampus (p<0.05 FWE SVC; top right panel). (B) Helicopter view of the circular environment that participants (black arrow) explored to approach the flower (red trace). For flowers in the danger zone, there was greater activation in the dorsomedial prefrontal cortex (dmPFC) across the whole test session in ANX compared to HC (p<0.05 FWE SVC; lower right panel). All images are presented at p<0.001 uncorrected for display purposes, not all clusters shown are significant at the whole-brain FWE corrected level used outside of our ROIs. Percentage signal changes, presented in bar graph and scatter plot for individual data points, for danger and safety across early and late periods of learning extracted from (A) sACC (MNI coordinates: 5, 9, -11; left panel) and anterior hippocampus (MNI coordinates: -27, -17, -14; right panel); and (B) dmPFC (MNI coordinates: 9, 26, 45; right panel). Only a subset of percentage signal change graphs are shown to illustrate the pattern of activation, similar pattern of activation were observed in the other relevant areas per contrast. Error bars show the standard error mean. *p<0.05 FWE SVC.*

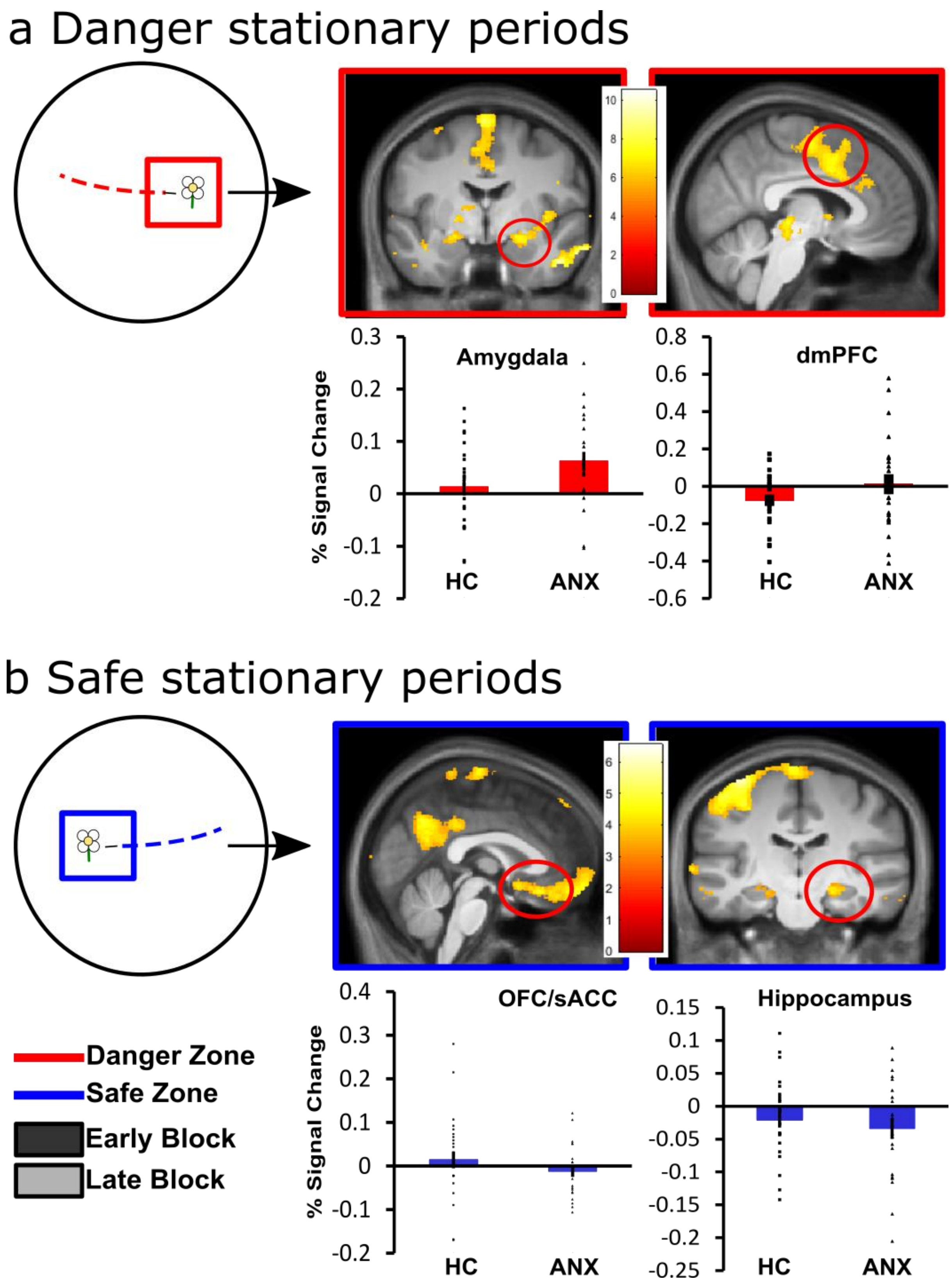

a Danger stationary periods
10
8
6
4
2
0
Amygdala
dmPFC
% Signal Change
0.3
0.2
0.1
0
-0.1
-0.2
0.8
0.6
0.4
0.2
0
-0.2
-0.4
-0.6
HC
ANX
HC
ANX
b Safe stationary periods
6
5
4
3
2
1
0
OFC/sACC
Hippocampus
% Signal Change
0.4
0.3
0.2
0.1
0
-0.1
-0.2
0.15
0.1
0.05
0
-0.05
-0.1
-0.15
-0.2
-0.25
HC
ANX
HC
ANX
Danger Zone
Safe Zone
Early Block
Late Block

Supplementary Figure 2*. fMRI results of stationary periods.* *Circular illustrations: Helicopter view of the circular environment that participants explored. The stationary period is represented for the dangerous flower as a red square and for the safe flower as a blue square. (A) The stationary period in the dangerous showed greater activation in the periaqueductal gray, dorsomedial prefrontal cortex (dmPFC), dorsal anterior cingulate cortex (dACC; middle panel), amygdala, and insula ($p<0.05$ FWE; right panel) in ANX. (B) The stationary period in the safe zone showed greater activation in the posterior cingulate cortex (PCC; $p<0.05$ FWE), ventromedial prefrontal cortex (vmPFC), orbitofrontal cortex/subgenual anterior cingulate cortex (OFC/sACC; lower middle panel), and anterior hippocampus ($p<0.05$ FWE SVC; lower right panel) in HC. All images are presented at $p<0.001$ uncorrected for display purposes, not all clusters shown are significant at the whole-brain FWE corrected level used outside of our ROIs. Percentage signal changes, presented in bar graph and scatter plot for individual data points, during stationary periods for danger and safety across early and late parts of learning extracted from (A) amygdala (MNI coordinates: 26, -2, -15; middle panel) and dmPFC (MNI coordinates: 0, -8, 71; right panel) and (B) OFC (MNI coordinates: -3, 54, -17; middle panel) and anterior hippocampus (MNI coordinates: -32, -29, 12; right panel). Only a subset of percentage signal change graphs are shown to illustrate the pattern of activation, similar pattern of activation were observed in the other relevant areas per contrast. Error bars show the standard error mean, **$p<0.05$ FWE; *$p<0.05$ FWE SVC.*

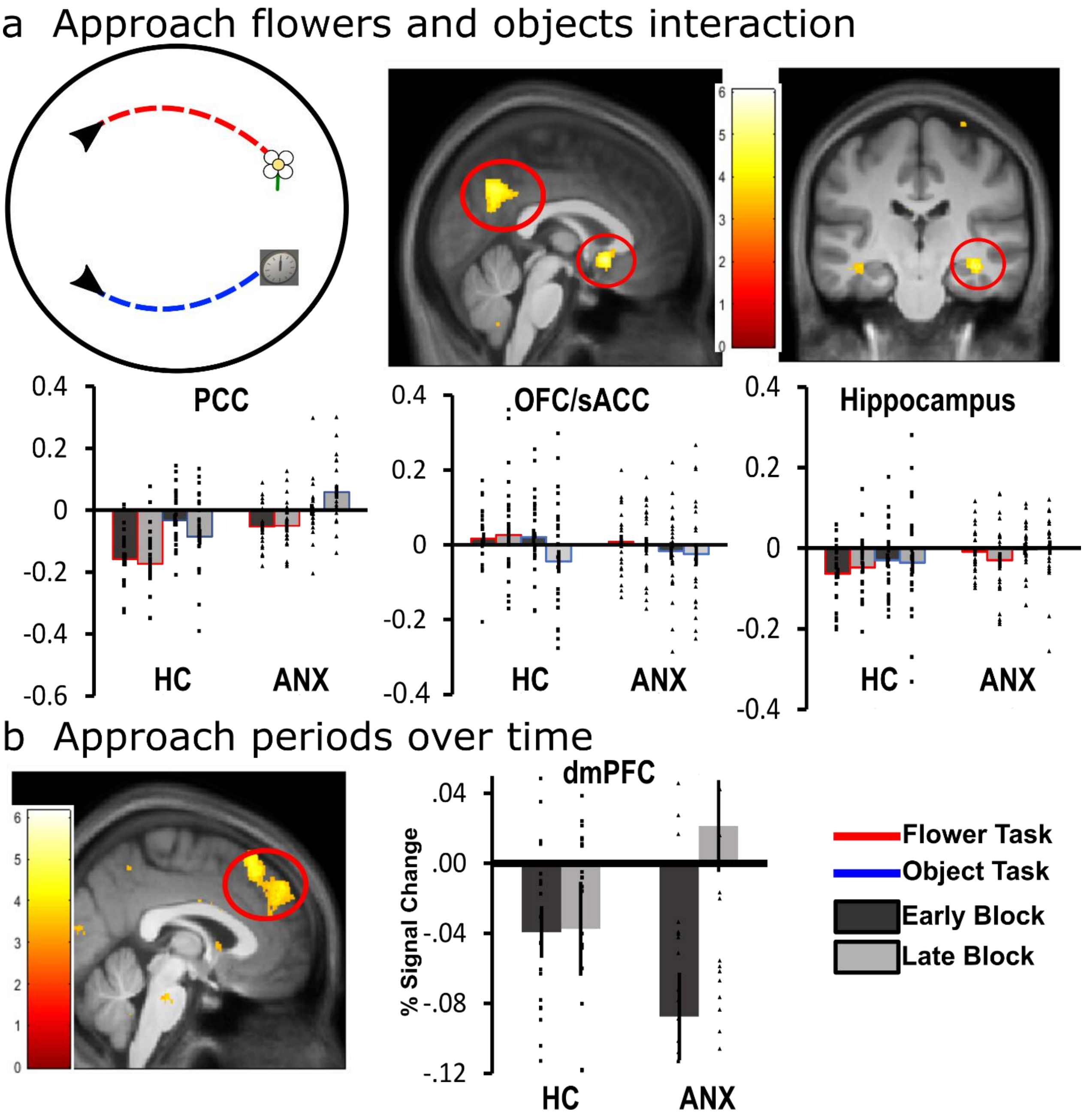

Supplementary Figure 3. Activation differences between approaching flowers and objects during threat and spatial memory. *(A) Circular illustration (left panel): helicopter view of the circular environment that participants (black arrow) explored and approach to emotional-flower (red-trace) and approach to unemotional object (blue-trace). Percentage signal change of task by block by group interaction when approaching flowers compared to objects in a range of brain areas, including the posterior cingulate cortex (PCC; p<0.05 FWE; middle panel), orbitofrontal*

*cortex/subgenual anterior cingulate cortex (OFC/sACC; $p<0.05$ FWE; middle panel), and hippocampus ($p<.0.05$ FWE SVC; right panel), (B) Activation change was greater from the first to the second half experiment (late > early) in the dorsomedial prefrontal cortex (dmPFC) in ANX ($p<0.05$ FWE SVC; left panel). All images are presented at $p<0.001$ uncorrected for display purposes, not all clusters shown are significant at the whole-brain FWE corrected level used outside of our ROIs. Percentage signal changes, presented in bar graph and scatter plot for individual data points, for learning about threat and object locations across early and late periods of the task extracted from (A) PCC (MNI coordinates: -2, -63, 21; left panel), sACC (MNI coordinates: 0, 14, -8; middle panel), hippocampus (MNI coordinates: 23, -20, -14; right panel), and (B) dmPFC (MNI coordinates: 0, 29, 53; middle panel). Only a subset of percentage signal change graphs are shown to illustrate the pattern of activation, similar pattern of activation were observed in the other relevant areas per contrast. Error bars show standard error mean, **$p<0.05$ FWE, *$p<0.05$ FWE SVC.*

Supplementary tables

Supplementary Table 1. Summary of between-group imaging findings when approaching flowers predicting danger or safety.

Region	Laterality	MNI coordinates x	y	z	z-score
**<u>Interaction threat (danger, safety) x block (early, late) x group (ANX, HC)</u>**					
Orbitofrontal Cortex/subgenual anterior cingulate cortex	R	5	9	-11	4.84
	R	5	29	-12	4.58
Hippocampus	R	23	-17	-18	4.62
	L	-27	-17	-14	4.02
Ventromedial Prefrontal Cortex	R	3	51	14	4.60
Posterior Cingulate Cortex†	L	-11	-54	21	4.28
**<u>Interaction threat (danger > safe) x group (ANX > HC)</u>**					
Insula†	L	-44	15	0	4.01
	R	41	20	3	3.33
Dorsomedial Prefrontal Cortex	R	9	26	45	3.95
**<u>Interaction block (late > early) x group (HC > ANX)</u>**					
Hippocampus	R	26	-29	-8	3.04
Ventromedial Prefrontal Cortex	L	-9	50	-18	2.67
Orbitofrontal Cortex	R	9	39	-12	2.52

p<0.05 FWE SVC unless stated; †p<0.05 FWE across whole brain; ANX, patients with with pathological anxiety; HC, healthy controls

Supplementary Table 2. Summary of between-group imaging findings during freezing periods for flowers during danger and safety.

Region	Laterality	MNI coordinates x	y	z	z-score
**Interaction threat (danger, safety) x block (early, late) x group (ANX, HC)**					
Ventromedial Prefrontal Cortex	R	12	47	2	4.31
	L	-9	59	9	3.73
Posterior Cingulate Cortex†	R	6	-50	18	3.96
Orbitofrontal Cortex	L	-5	15	-9	3.94
	L	-5	36	-12	3.94
**Interaction threat (danger > safety) x group (ANX > HC)**					
Dorsomedial Prefrontal Cortex†		0	-8	71	4.68
	R	8	18	51	4.63
	R	6	38	24	4.51
Insula†	L	-39	21	-5	4.44
	R	41	-6	0	4.20
Amygdala†	R	26	-2	-15	4.13
	L	-20	2	-15	4.10
Periaqueductal Grey†	R	6	-26	-5	4.08
	L	-2	-29	-8	3.37
Caudate†	L	-9	9	3	4.03

	R	11	9	2	3.63
Dorsal Anterior Cingulate Cortex┼	L	-3	25.5	21	3.84
Thalamus┼	R	11	-8	8	3.77
	L	-9	9	3	3.30
**Interaction threat (safety > danger) x group (HC > ANX)**					
Posterior Cingulate Cortex┼	L	-3	-60	27	4.66
Orbitofrontal Cortex/subgenual anterior cingulate cortex	L	-3	54	-17	4.64
	R	2	11	-12	4.03
Hippocampus	R	23	-18	-14	4.08
	L	-32	-29	-12	3.85
Ventromedial Prefrontal Cortex	L	-3	65	14	3.24
**Interaction block (late > early) x group (ANX > HC)**					
Dorsomedial Prefrontal Cortex	R	3	32	44	3.65
**Interaction block (late > early) x group (HC > ANX)**					
Orbitofrontal Cortex	R	14	44	-15	4.62
Ventromedial Prefrontal Cortex	R	12	54	-3	3.92
	R	11	47	17	3.72
Posterior Cingulate Cortex	R	3	-41	26	2.80

p<0.05 FWE SVC unless stated; ┼p<0.05 FWE across whole brain; ANX, patients with pathological anxiety; HC, healthy controls

Supplementary Table 3. Summary of between-group imaging findings when approaching flowers during threat learning or objects during spatial memory encoding.

Region	Laterality	MNI coordinates x	y	z	z-score
**<u>Interaction Task (flower, object) x block (early, late) x group (ANX, HC)</u>**					
Posterior Cingulate Cortex┼	L	-2	-63	21	3.80
Orbitofrontal Cortex/subgenual anterior cingulate cortex		0	14	-8	3.28
Hippocampus	R	23	-20	-14	3.13
**Interaction block (late > early) x group (ANX > HC)**					
Dorsomedial Prefrontal Cortex		0	29	53	4.44
**Interaction task (flower > object) x group (HC > ANX)**					
Orbitofrontal Cortex	L	-5	32	-20	3.58

p<0.05 FWE SVC unless stated; ┼p<0.05 FWE across whole brain; ANX, patients with pathological anxiety; HC, healthy controls

Supplementary Table 4: Demographic information of the sample

		<u>HC</u>	<u>ANX</u>
**N**		28	23
**Sex, N (%)**	Male	16 (57.1)	12 (52.2)
	Female	12 (42.9)	11 (47.8)
**Race, N (%)**	Black/African American	2 (7.1)	3 (13.0)
	White/Caucasian	13 (46.4)	16 (69.6)
	Asian	11 (39.3)	2 (8.7)
	Mixed/Other	2 (7.1)	2 (8.7)
**Age, mean years (SD)**		27.25 (8.2)	29.6 (8.3)
**STAI (pre-task), mean (SD)**		25.8 (6.9)	39.6 (9.8)
**STAI (post-task), mean (SD)**		26.0 (6.6)	41.7 (11.2)
	GAD	0	8 (34.8)
**Current (%)**	SAD	0	7 (30.4)
	GAD & SAD	0	8 (34.8)

ANX, patients with pathological anxiety; HC, healthy controls; GAD, Generalized anxiety disorder; SAD; social anxiety disorder.
