# Supplementary material for: Location-dependent threat and associated neural abnormalities in clinical anxiety
Source: Commun Biol. 2021 Nov 4;4:1263. doi: 10.1038/s42003-021-02775-x (PMC8568971; doi:10.1038/s42003-021-02775-x)
Supplement: Supplementary file 3 — Reporting Summary [file 42003_2021_2775_MOESM3_ESM.pdf]

nature portfolio

Corresponding author(s): Benjamin Suarez-Jimenez

Last updated by author(s): September 27, 2021

Reporting Summary

Nature Portfolio wishes to improve the reproducibility of the work that we publish. This form provides structure for consistency and transparency in reporting. For further information on Nature Portfolio policies, see our Editorial Policies and the Editorial Policy Checklist.

Statistics

For all statistical analyses, confirm that the following items are present in the figure legend, table legend, main text, or Methods section.

n/a	Confirmed	
☐	☒	The exact sample size (*n*) for each experimental group/condition, given as a discrete number and unit of measurement
☐	☒	A statement on whether measurements were taken from distinct samples or whether the same sample was measured repeatedly
☐	☒	The statistical test(s) used AND whether they are one- or two-sided<br>*Only common tests should be described solely by name; describe more complex techniques in the Methods section.*
☐	☒	A description of all covariates tested
☐	☒	A description of any assumptions or corrections, such as tests of normality and adjustment for multiple comparisons
☐	☒	A full description of the statistical parameters including central tendency (e.g. means) or other basic estimates (e.g. regression coefficient) AND variation (e.g. standard deviation) or associated estimates of uncertainty (e.g. confidence intervals)
☐	☒	For null hypothesis testing, the test statistic (e.g. *F*, *t*, *r*) with confidence intervals, effect sizes, degrees of freedom and *P* value noted<br>*Give P values as exact values whenever suitable.*
☒	☐	For Bayesian analysis, information on the choice of priors and Markov chain Monte Carlo settings
☐	☒	For hierarchical and complex designs, identification of the appropriate level for tests and full reporting of outcomes
☐	☒	Estimates of effect sizes (e.g. Cohen's *d*, Pearson's *r*), indicating how they were calculated

Our web collection on statistics for biologists contains articles on many of the points above.

Software and code

Policy information about availability of computer code

Data collection	Blood oxygen level-dependent T2*-weighted functional images were acquired on a 3T Skyra system (Siemens, Germany) using echo-planar imaging (EPI) with a 32-channel head coil. Images were acquired with a 45° oblique angle with the following parameters: 3300 ms TR; 30 ms TE; 1 mm inter-slice gap, 192 mm field of view, and 48 axial slices with 2 mm slice thickness resulting in 3mm isotropic voxels. A single echo field map was recorded for distortion correction of the acquired EPI. After the functional scans, a T1-weighted 3-D structural image (1mm3) was acquired to coregister and display the functional data.<br>Skin conductance was measured as an index of anxiety via 8mm Ag/AgCl electrodes attached to the medial phalanges of the index and middle fingers of the participant's left hand. Data were acquired using a Biopac EDA100C MRI system (Biopac Systems, Inc., Goleta, CA, USA) at a sampling rate of 1000Hz.<br>The virtual environment task was developed with Unity Software (Unity Technologies, USA).
Data analysis	Data processing and analysis were performed using SPM12 (http://www.fil.ion.ucl.ac.uk/spm). Data processing and analysis of electrodermal activity (EDA) and behavioral analysis were performed using MATLAB.

For manuscripts utilizing custom algorithms or software that are central to the research but not yet described in published literature, software must be made available to editors and reviewers. We strongly encourage code deposition in a community repository (e.g. GitHub). See the Nature Portfolio guidelines for submitting code & software for further information.

Data

Policy information about availability of data

All manuscripts must include a data availability statement. This statement should provide the following information, where applicable:

- Accession codes, unique identifiers, or web links for publicly available datasets
- A description of any restrictions on data availability
- For clinical datasets or third party data, please ensure that the statement adheres to our policy

The datasets generated during and/or analyzed during the current study will be made available in neurovault and referenced in publication.

Field-specific reporting

Please select the one below that is the best fit for your research. If you are not sure, read the appropriate sections before making your selection.

☐ Life sciences ☒ Behavioural & social sciences ☐ Ecological, evolutionary & environmental sciences

For a reference copy of the document with all sections, see nature.com/documents/nr-reporting-summary-flat.pdf

Behavioural & social sciences study design

All studies must disclose on these points even when the disclosure is negative.

Study description	In fMRI, patients with pathological anxiety (ANX, n=23) and healthy controls (HC, n=28) completed a contextual threat learning paradigm in which they picked flowers in a virtual environment comprising a danger zone in which flowers were paired with shock and a safe zone (no shock). The study aimed at elucidating neural mechanisms of learning about safe and danger within an environment and how it differs in pathological anxiety.
Research sample	Sixty participants, aged 18-50 years, were recruited from the Washington D.C. and Maryland areas. Thirty participants were diagnosed with an anxiety disorder (ANX; generalized anxiety disorder (n=8), social anxiety disorder (n=7), and co-morbidity between the two (n=8)) using the Structured Clinical Interview for Diagnostic and Statistical Manual of Mental Disorders 4th edition, while the other 30 volunteers were healthy controls (HC).
Sampling strategy	At the time of the conception of the study (2014), a sample size of 15 subjects with good quality data (no major artifacts or other problems) was usual for previous published studies on activation of limbic structures, such as Phelps et al. (1999; N=12), and Whalen et al. (1998; N=10). Although power computations were possible for ROI analyses using conventional statistical tests in which average BOLD signal intensities are treated as dependent variables, because the study constituted an experiment that had not been previously performed, the mean and standard deviation of the changes in BOLD responses that were obtained using the proposed methods were not known at the time of designing the study. In general, we thought with sample sizes as small as n=10 and for alpha = .05 (two-tailed), power exceeds .80 to detect effect sizes of 1.2 in one pre-specified brain region. Fortunately, most fMRI studies of neurobiologically meaningful task-associated changes in BOLD signal document larger effects than this. For patients (i.e., GAD, SAD, panic, and PTSD) an N of 30 was targeted for the study. This larger sample size would provide us with a greater range of symptom severity in order to conduct correlation analyses with regional brain activity and it would buffer against potentially higher rates of attrition (excessive motion, symptom-related dropout) in our psychiatric samples.
Data collection	Skin conductance. Skin conductance was measured as an index of anxiety via 8mm Ag/AgCl electrodes attached to the medial phalanges of the index and middle fingers of the participant's left hand. Data were acquired using a Biopac EDA100C MRI system (Biopac Systems, Inc., Goleta, CA, USA) at a sampling rate of 1000Hz.<br>Shocks. Shocks (20 samples) were applied using a Digitimer DS7A electrical stimulator (Digitimer, Welwyn Garden City, UK) to the left hand with intensity up to 50mA for 2ms duration through a silver chloride electrode. Shock intensity was adjusted individually for each participant before starting the experiment. Individual adjustment procedures delivered a series of shocks to each participant, starting at 12mA. Participants were asked to rate the level of pain with each shock on a 1-10 scale. Shock intensity was increased until the level was irritating but not painful.<br>Task. A description of the virtual environment task, developed with Unity Software (Unity Technologies, USA), is available in a prior publication.<br>fMRI acquisition. Blood oxygen level-dependent T2*-weighted functional images were acquired on a 3T Skyra system (Siemens, Germany) using echo-planar imaging (EPI) with a 32-channel head coil. Images were acquired with a 45° oblique angle with the following parameters: 3300 ms TR; 30 ms TE; 1 mm inter-slice gap, 192 mm field of view, and 48 axial slices with 2 mm slice thickness resulting in 3mm isotropic voxels. A single echo field map was recorded for distortion correction of the acquired EPI. After the functional scans, a T1-weighted 3-D structural image (1mm3) was acquired to coregister and display the functional data.
Timing	Data was collected from 2015-2017 at the NIMH.
Data exclusions	Four ANX were excluded from analyses because they were unable to explain the shock contingencies between the locations at the end of the task (see procedure below). Three ANX and two HC were omitted due to excessive head motion (>20%; see below). Therefore, the final sample included 23 ANX (mean age=29.61; SD=8.22) and 28 HC (mean age=27.25; SD=8.21)
Non-participation	No participants dropped out or declined participation.

Randomization	Participants were divided by anxiety diagnosis, all participants regardless of anxiety diagnosis performed the same protocol and task.

Reporting for specific materials, systems and methods

We require information from authors about some types of materials, experimental systems and methods used in many studies. Here, indicate whether each material, system or method listed is relevant to your study. If you are not sure if a list item applies to your research, read the appropriate section before selecting a response.

Materials & experimental systems

n/a	Involved in the study
☒	☐ Antibodies
☒	☐ Eukaryotic cell lines
☒	☐ Palaeontology and archaeology
☒	☐ Animals and other organisms
☐	☒ Human research participants
☒	☐ Clinical data
☒	☐ Dual use research of concern

Methods

n/a	Involved in the study
☒	☐ ChIP-seq
☒	☐ Flow cytometry
☐	☒ MRI-based neuroimaging

Human research participants

Policy information about studies involving human research participants

Population characteristics	Age, sex, race, past and current anxiety diagnosis were considered in this study.
Recruitment	Participants were recruited from the Washington D.C. and Maryland areas through advertisements in local media, bulletin boards, and Internet ads.
Ethics oversight	The study was approved by the NIH Institutional Review Board.

Note that full information on the approval of the study protocol must also be provided in the manuscript.

Magnetic resonance imaging

Experimental design

Design type	Free-behavior design, task based fMRI.
Design specifications	Participants freely played a virtual reality task while their brain activity was constantly recorded. The task included a total of 80 flowers—40 flowers were paired with a shock on 50% of the trials (danger) while encountering no shock with the remaining 40 (safe).
Behavioral performance measures	When participants picked a flower, they rated on a scale from 0 to 9 the likelihood of receiving a shock from the flower picked. Each rating (0-9) was averaged across trials to create four equal blocks separated by safe and danger conditions (10 trials in each block).<br>After every four flower trials, we included a spatial memory trial in which participants learned the location of one of four objects (wooden box, gas can, book, and clock); however, two objects appeared in each half of the environment. No shocks accompanied spatial memory stimuli. After the first four spatial memory trials, participants' memory for object locations was tested by asking participants to replace the objects where they originally found them.Performance on the spatial memory task was analyzed by assessing distance error on each test trial. This distance error was calculated by taking the distance in virtual meters between the participant's response location when replacing the object and its correct location within the environment. Distance error was taken from each trial and averaged into four blocks (1 trial from each object in each block).<br>At the end of the experiment, participants were asked to name the four objects and their locations, as well as explain the contingencies of danger and safety during threat learning, by answering if there was a pattern in the shocks. Participants who were unable to provide the objects' name and position or explain the contingencies were excluded from the final analysis to ensure that participants were paying attention to the task.

Acquisition

Imaging type(s)	functional
Field strength	3T
Sequence & imaging parameters	Blood oxygen level-dependent T2*-weighted functional images were acquired on a 3T Skyra system (Siemens, Germany) using echo-planar imaging (EPI) with a 32-channel head coil. Images were acquired with a 45° oblique angle with the following parameters: 3300 ms TR; 30 ms TE; 1 mm inter-slice gap, 192 mm field of view, and 48 axial slices with 2 mm

slice thickness resulting in 3mm isotropic voxels. A single echo field map was recorded for distortion correction of the acquired EPI. After the functional scans, a T1-weighted 3-D structural image (1mm3) was acquired to coregister and display the functional data.

Area of acquisition: Whole brain

Diffusion MRI: ☐ Used ☒ Not used

Preprocessing

Preprocessing software: Data processing and analysis were performed using SPM12 (http://www.fil.ion.ucl.ac.uk/spm).

Normalization: Images were then spatially normalized to the MNI template using parameter estimates from warping each participant's structural image to a T1-weighted average template image.

Normalization template: Images were then spatially normalized to the MNI template using parameter estimates from warping each participant's structural image to a T1-weighted average template image.

Noise and artifact removal: EPI images were first preprocessed using a bias correction to control for within volume signal intensity difference, unwarping and realignment to correct for movement and slice-time correction. Six regressors of no interest were also added to the model representing movement parameters estimated during realignment. Frames with more than 0.5 mm frame-wise head motion were detected as outliers and modeled using the Artifact detection tool (ART). Participants with outliers totaling 20% or more of their total scan were removed from analysis.

Volume censoring: Frames with more than 0.5 mm frame-wise head motion were detected as outliers and modeled using the Artifact detection tool (ART). Participants with outliers totaling 20% or more of their total scan were removed from analysis.

Statistical modeling & inference

Model type and settings: The analysis model included 15 regressors of interest. Four separate regressors of interest were created for approach periods by zone (safe or danger) and block (first or second half of experiment); these consisted of boxcar functions from the end of the first quarter of each approach period to the point in which the flower was reached. Four regressors of interest (also by zone and block) were created for the stationary period of each trial, consisting of a boxcar function starting after the participant rated their shock expectancy and lasting for the duration of the stationary period. Three regressors using a stick/impulse function modeled the end of each trial in safe conditions and in danger conditions split by whether participants received a shock or not. Finally, four regressors were modeled when participants were replacing objects during the spatial memory task using a boxcar function covering when a response was made and the approach period to the location where the object was picked (first and second half of the experiment). Six regressors of no interest were also added to the model representing movement parameters estimated during realignment.

Effect(s) tested: Statistical analyses occurred in two stages, individual (general linear model analysis) and group level (two sample t-test). At the individual level, an initial control analysis was conducted to examine emotional flower vs. unemotional object approaches to a cue. The neural responses to approach periods were compared between the threat learning task (approaching flowers) and the spatial memory task (approaching location to replace the object; see supplementary material). We created a model contrasting approach periods for threat learning (collapsing across safety and danger) with approach during spatial memory across the first and second half of the experiment using a 2x2 ANOVA (task, block). Approach periods during threat learning were then examined by contrasting approach to flowers associated with safety or danger and collected during the first or second half of the experiment using a 2x2 ANOVA (zone, block). Finally, stationary periods during threat learning, periods when the flower was picked, and participants were held stationary were analyzed in a similar first-level analysis using a 2x2 ANOVA (zone, block).
At the group level, the contrasts of interest, described above, were entered into two-sample t-tests to compare the ANX and HC. Within this model, t-contrasts were examined to investigate ANX>HC and HC>ANX differences. To aid and confirm interpretation of significant group effects, parameter estimates were extracted from significant peak coordinates for the contrasts ANX>HC and HC>ANX using the MarsBaR region of interest toolbox. Post-hoc analysis of these contrasts was conducted using repeated measures 2x2x2 ANOVA (zone or task, block, group) in SPSS.

Specify type of analysis: ☐ Whole brain ☐ ROI-based ☒ Both

Anatomical location(s): ROI approach was employed based on the Automated Anatomical Labeling atlas (AAL)38 and the WFU Pickatlas toolbox in SPM12.39 One bilateral mask was defined which included the hippocampus, amygdala, and mPFC (orbitofrontal gyrus, ventromedial prefrontal cortex, dorsomedial prefrontal cortex, and anterior cingulate and medial cingulate cortex). Statistical threshold in this single mask was defined by small volume correction (SVC; p<0.05 FWE).

Statistic type for inference (See Eklund et al. 2016): Voxelwise-based family-wise error (FWE; p<0.05) corrected effects across the whole brain are reported for all analyses.

Correction: FWE

Models & analysis

n/a	Involved in the study
☐	☒ Functional and/or effective connectivity
☒	☐ Graph analysis
☒	☐ Multivariate modeling or predictive analysis

Functional and/or effective connectivity	Functional connectivity was assessed at the individual level using psychophysiological interaction (PPI) analysis with SPM12. The PPI compares functional connectivity from a single seed region across multiple task conditions. For exploratory analyses, seed regions were selected based on group differences identified in the main analysis (dmPFC, amygdala). Peak activation from these brain areas in the group level analysis, for approach and stationary periods, was used to create regions of interest (6 mm sphere centered on group level peak activation) for each participant. The seed time series activation for each participant was extracted at the center of the activation peak. The individual t-contrast images of the interaction from the PPI were examined using a group level one-sample t-test. As in a previous study,8 and to increase the strength and precision of the analysis, the PPIs were detected using a t-test with a threshold of p<0.001 corrected for multiple comparisons.
